# Synergistic anticancer activity of cisplatin combined with tannic acid enhances apoptosis in lung cancer through the PERK-ATF4 pathway

**DOI:** 10.1186/s40001-023-01420-z

**Published:** 2023-10-27

**Authors:** Xiang Zheng, Lei Yang, Wei Zhai, Nana Geng, Zhimin Zhang, Xueying Li, Mingsong Wu

**Affiliations:** 1 Department of Genetics, Zunyi Medical University, Xinpu Campus, No. 6, Xuefu West Road, Xinpu New District, Zunyi, Guizhou China; 2 School of Stomatology, Zunyi Medical University, Xinpu Campus, No. 6, Xuefu West Road, Xinpu New District, Zunyi, Guizhou China; 3 Special Key Laboratory of Oral Disease Research and High Education Institute in Guizhou Province, School of Stomatology, Zunyi Medical University, Zunyi, Guizhou China; 4 Qihe County Vocational Secondary Professional School, Dezhou, Guizhou China

**Keywords:** Lung cancer, Endoplasmic reticulum stress, Tannins, Cisplatin, Combination drugs

## Abstract

**Background:**

Cisplatin (CDDP) is a common anticancer drug whose side effects limit its clinical applications. Tannins (TA) are plant-derived polyphenols that inhibit tumor growth in different types of cancer. Here, we evaluated the anticancer effect of TA combined with CDDP on lung cancer cell lines (GLC-82 and H1299) and investigated the underlying molecular mechanism of endoplasmic reticulum (ER) stress-induced apoptosis.

**Methods:**

Cell lines were treated with CDDP, TA, and CDDP + TA, and the effect of the combination was assessed using MTT assay and observed under light and fluorescence microscopes. Cell apoptosis was detected by flow cytometry, and the levels of ERS apoptosis pathway related genes were valuated by qRT-PCR and western blotting. The effects of the drug combination on the tumors of nude mice injected with H1299 cells were investigated, and the expression of key factors in the ER stress apoptotic pathway was investigated.

**Results:**

The combination of CDDP and TA significantly inhibited lung cancer cell viability indicating a synergistic antitumoral effect. The mRNA and protein expression levels of key ER stress factors in the CDDP + TA group were considerably higher than those in the CDDP and TA groups, the tumor volume in tumor-bearing mice was the smallest, and the number of apoptotic cells and the protein expression levels of the key ER stress in the combination group were considerably higher.

**Conclusions:**

The combination of TA and CDDP may produce synergistic antitumoral effects mediated by the PERK-ATF4-CHOP apoptotic axis, suggesting a novel adjuvant treatment for lung cancer.

## Introduction

Despite advances in the treatment and early diagnosis of lung cancer, it remains the leading cause of cancer-related deaths worldwide [1]. Non-small cell lung cancer (NSCLC) accounts for 85% of lung cancer cases, and most patients with NSCLC are often diagnosed at an advanced stage with metastasis and not fit to perform a surgical operation [2]. Currently, platinum-based chemotherapies remain the standard treatment for lung cancer [3, 4].

Cisplatin (*cis*-diamminedichloroplatinum or CDDP), a platinum-based drug, is a commonly used and highly effective anticancer drug against various cancers, including lung cancer [5]. However, its clinical application is limited owing to its low bioavailability, high toxicity, and frequent occurrence of drug resistance [6, 7]. Although dose increase is a common option, CDDP exhibits dose-dependent toxicity [8]. Clinical research has gradually shifted from monotherapy to combination therapy to achieve synergetic effects [9, 10]. CDDP combination therapy has gained increasing attention, aiming to reduce the side effects of CDDP monotherapy [11, 12]. Moreover, several low-toxicity natural products can enhance the anticancer effects of platinum-based drugs [13, 14].

Tannic acid (TA), found in a variety of common foods, including nuts, beans, and grapes, is a plant-derived polyphenolic compound. TA can enhance the effects of various hydrophobic drugs such as curcumin, rapamycin, and paclitaxel [15]. TA can induce cell cycle arrest and apoptosis through the TRAIL-mediated exogenous apoptotic pathway and regulate mitochondrial reactive oxygen species (mROS) to induce exogenous apoptosis in human embryonic cancer cells [16]. It can also promote apoptosis through the endoplasmic reticulum (ER) stress-mediated unfolded protein response (UPR) pathway [17]. Additionally, TA can reduce the cell viability of breast cancer cells, increase the expression of p53 in breast tumors, enhance the anticancer activity of doxorubicin, and inhibit the cardiotoxicity induced by doxorubicin [18, 19]. Therefore, TA can be used as a monomeric and adjuvant anticancer drug.

The combination of TA and cisplatin induces apoptosis in ovarian and liver cancer cells [20, 21]. The binding of TA to lung fluid depends on the adsorption of lung fluid, which reduces the surface tension and thereby enhances the interaction between TA and lung cancer cells [22]. Therefore, TA can be used as a carrier of carboplatin and other drugs and improve the bioavailability and targeting of active drugs for lung cancer treatment. However, whether TA can synergistically enhance the antitumoral effects of CDDP in lung cancer remains unclear. Hence in this study, we focused the synergistic effect of TA and CDDP on lung cancer through the ER stress pathway.

## Methods

### Cell culture

The human lung cancer cell line NCI-H1299 was obtained from the Cell Bank of the Chinese Academy of Sciences. The GLC-82 cell line was a gift from Dr. Cao Yi of the Kunming Institute of Zoology, Chinese Academy of Sciences. Cells were cultured in RPMI-1640 medium (Gibco; Thermo Fisher Scientific, Waltham, MA, USA) supplemented with 10% fetal bovine serum (Zhejiang TianHang Biological Science and Technology Co., Ltd., China), 100 U/mL penicillin, and 100 μg/mL streptomycin and incubated at 37 ℃ and 5% CO_2_. Cells with exponential growth were used in this study.

### Cell viability assay

H1299 and GLC-82 cells (1 × 10^4^ cells/well) were plated in 96-well plates and incubated for 24 h. CDDP (Qilu Pharmaceutica, Shandong, China) and TA (Thermo Fisher Scientific) were added to 96-well plates according to the present concentration and incubated for 24 h. After treatment with 10 μL MTT (Solarbio Life Sciences, Beijing, China) for 2 h, the supernatant was aspirated, and 100 μL of DMSO (Solarbio Life Sciences) was added to each well. The absorbance of each well was determined at 570 nm using a microplate reader (Spectra Max Mze; Molecular Devices, San Jose, CA, USA). The cell inhibition rate was calculated as follows:

$$\mathrm{inhibition\, rate}(\mathrm{\%})=1-(\mathrm{drug \,A \,value}-\mathrm{control \,A \,value})/(\mathrm{control \,A \,value}-\mathrm{blank \,A \,value})*100\mathrm{\%}$$

### Determination of combination and dose reduction indexes

The combination index (CI) and dose reduction index (DRI) are commonly used methods for determining the synergistic effect between two drugs and were calculated as follows:

$$\mathrm{CI}=(\mathrm{D})1/(\mathrm{Dx})1+(\mathrm{D})2/(\mathrm{Dx})2;\,(\mathrm{DRI})1=(\mathrm{Dx})1/(\mathrm{D})1, (\mathrm{DRI})2=(\mathrm{Dx})2/(\mathrm{D})2,$$

where (Dx) 1 and (Dx) 2 represent the doses of drugs 1 and 2 required to inhibit cell growth by 50%, respectively, and (D) 1 and (D) 2 indicate the individual doses of the two drugs required for 50% inhibition of cell growth by the combination of drugs. The combined effects of the two drugs can be indicated as follows:

$$\mathrm{CI}<1\,(\mathrm{cooperative\,effect}),\,\mathrm{CI}=1 (\mathrm{additive\,effect}),\,\mathrm{and}\,\mathrm{CI}>1(\mathrm{antagonistic\,effect}).$$

The higher the DRI value, the lower the drug dosage in combination with a single drug for the same efficacy.

### DAPI (4′,6-diamidino-2-phenylindole) staining

H1299 and GLC-82 cells (3 × 10^5^ cells/well) were plated in 6-well plates. According to CI and DRI, H1299 cells were divided into control (equal amount of medium), CDDP (7.5 μg/mL), TA (85 μg/mL), and CDDP (7.5 μg/mL) + TA 85 μg/mL) groups. GLC-82 cells were divided into control (equal amounts of medium), CDDP (2.5 μg/mL), TA (298 μg/mL), and CDDP (2.5 μg/mL) + TA (298 μg/mL) groups. After treatment for 24–48 h, the morphological changes of the cells and nuclei were observed under an inverted fluorescence microscope (IX73; Olympus, Tokyo, Japan).

### Apoptosis detection assay

Annexin V-FITC/PI (Solarbio Life Sciences, Beijing, China) staining was used to identify apoptotic cells at different time points. Lung cancer cells were collected in Eppendorf tubes, washed twice with cold phosphate-buffered saline (PBS), and resuspended in a binding buffer. Annexin V-FITC (10 μL) and propidium iodide (PI) solutions (5 μL) were added to the cell suspension for 15 min. Flow cytometry was used to detect and analyze cell apoptosis within 1 h using a flow cytometer (Gallios; Beckman Coulter, Brea, CA, USA) and FlowJo software (version 7.6.3; Tree Star, Inc.).

### Animal studies

The animal experimental procedures used in this study were reviewed and approved by the Institutional Animal Use and Care Committee of Zunyi Medical University. Nude mice were purchased from the Guangdong Animal Center (Guangzhou, China). Mice were anesthetized by an intraperitoneal injection of 0.75% sodium pentobarbital (10 g/100 μL per body weight). Cells (1 × 10^7^) were suspended in 200 µL 0.9% saline and injected subcutaneously into the axilla of 6-week-old mice for 7 d. On the eighth day of inoculation, a tumor (the size of a rice grain) developed in the axillary region of the mice. The mice were then randomly divided into four groups: control, TA, CDD, and TA + CDD. The drugs were injected every other day (TA, 30 mg/kg; CDD, 5.0 mg/kg) for three weeks. Body weight, tumor length, and tumor width were measured every three days, and the tumor volume was calculated as follows:

$$\mathrm{V }= 1/2\times (\mathrm{length}\times \mathrm{width}2).$$

### Terminal deoxynucleotidyl transferase (TdT) dUTP Nick-End Labeling (TUNEL) assay

TUNEL analysis was performed using the Cell Apoptosis Detection Kit (Bio-Rad Laboratories, Hercules, CA, USA). TdT buffer was added to the tissue, incubated at 37 ℃ for 60 min, washed thrice with PBS, each time for 5 min. Slides were counterstained with DAPI for 5 min in the dark and washed with PBST four times for 5 min each. After the liquid on the slice was dried with absorbent paper, the film was collected, sealed, and observed under a fluorescence microscope. Apoptotic cells are stained red, and the nuclei are stained blue.

### Real-time quantitative PCR analysis (qRT-PCR)

Total RNA was isolated using the TRIzol method and reverse-transcribed into cDNA according to the preset steps. The real-time PCR reaction system (10 μL) comprised 1.0 μL, 5.0 μL ssofast evagreen supermix (Bio-Rad Laboratories, Shanghai, China), and 1 μL of primer. The reaction conditions were as follows: 94 ℃ 60 s, 95 ℃ 20 s, 56 ℃ (β-actin, GRP78) or 62.4 ℃ (ATF6) 30 s, 40 cycles. Three tubes were used for each sample. The relative expression of the gene was calculated using the 2^−△△CT^ method, with the β-actin as an internal reference and the average value of three replicates. Primers used for qPCR are listed in Table 1.

**Table 1:** Primers of genes related to endoplasmic reticulum stress pathway used in experiments

Gene	Primer sequence (5′-3′)	Length (bp)
*ATF4*	F:ATGACCGAAATGAGCTTCCTG R:GCTGGAGAACCCATGAGGT	153
*ATF6*	F:TCCTCGGTCAGTGGACTCTTA R:CTTGGGCTGAATTGAAGGTTTTG	235
*Caspase3*	F:GAAATTGTGGAATTGATGCGTGA R:CTACAACGATCCCCTCTGAAAAA	164
*CHOP*	F:CAAGAGGTCCTGTCTTCAGATGA R:TCTGTTTCCGTTTCCTGGTTC	247
*GAPDH*	F:AAGCTAGTTACAAAAAGGCCATCATT R:AGGGTTCGGACTCCTGGAA	45
*GRP78*	F:TCAAGTTCTTGCCGTTCAAGG R:AAATAAGCCTCAGCGGTTTCTT	148
*HSP90B1*	F:CTGGGACTGGGAACTTATGAATG R:TCCATATTCGTCAAACAGACCAC	217
*IRE1α*	F:CACAGTGACGCTTCCTGAAAC R:GCCATCATTAGGATCTGGGAGA	169
*PERK*	F:ACGATGAGACAGAGTTGCGAC R:ATCCAAGGCAGCAATTCTCCC	80
*EIf2α1*	F:GCTTGCTATGGTTACGAAGGC R: CATCACATACCTGGGTGGAG	120

Tips: F: forward primer; R: reverse primer

### Western blotting

Proteins were isolated using sodium dodecyl sulphate–polyacrylamide gel electrophoresis (SDS-PAGE) (generay biotechnology, Shanghai, China) and transferred to a polyvinylidene fluoride (PVDF) membrane. Membranes were incubated overnight at 4 ℃ with the following primary antibodies: HSP70 (1:1000), CHOP (1:1000), CASPASE3 (1:1000), PERK (1:400) (Abcam, Cambridge, UK), and ATF4 (1:500; Santa Cruz Biotechnology, Dallas, TX, USA). Membranes were then incubated for 2 h at 37 ℃ with the secondary antibodies (1:2000; ProteinTech Group, Wuhan, China;), then incubated with enhanced chemiluminescence (ECL), and the images were collected using an imaging system (Chembvc mp). Protein bands were analyzed using Image-Pro Plus software (version 6.0).

### Statistical analysis

The results are expressed as mean ± SD. SPSS 19.0 software was used to analyze the data. One-way way ANOVA was used to compare the results between the groups. Statistical significance was set at *P* < 0.05.

## Results

### TA enhances the growth inhibition effect of CDDP on H1299 and GLC82 cells

**Fig. 1:**
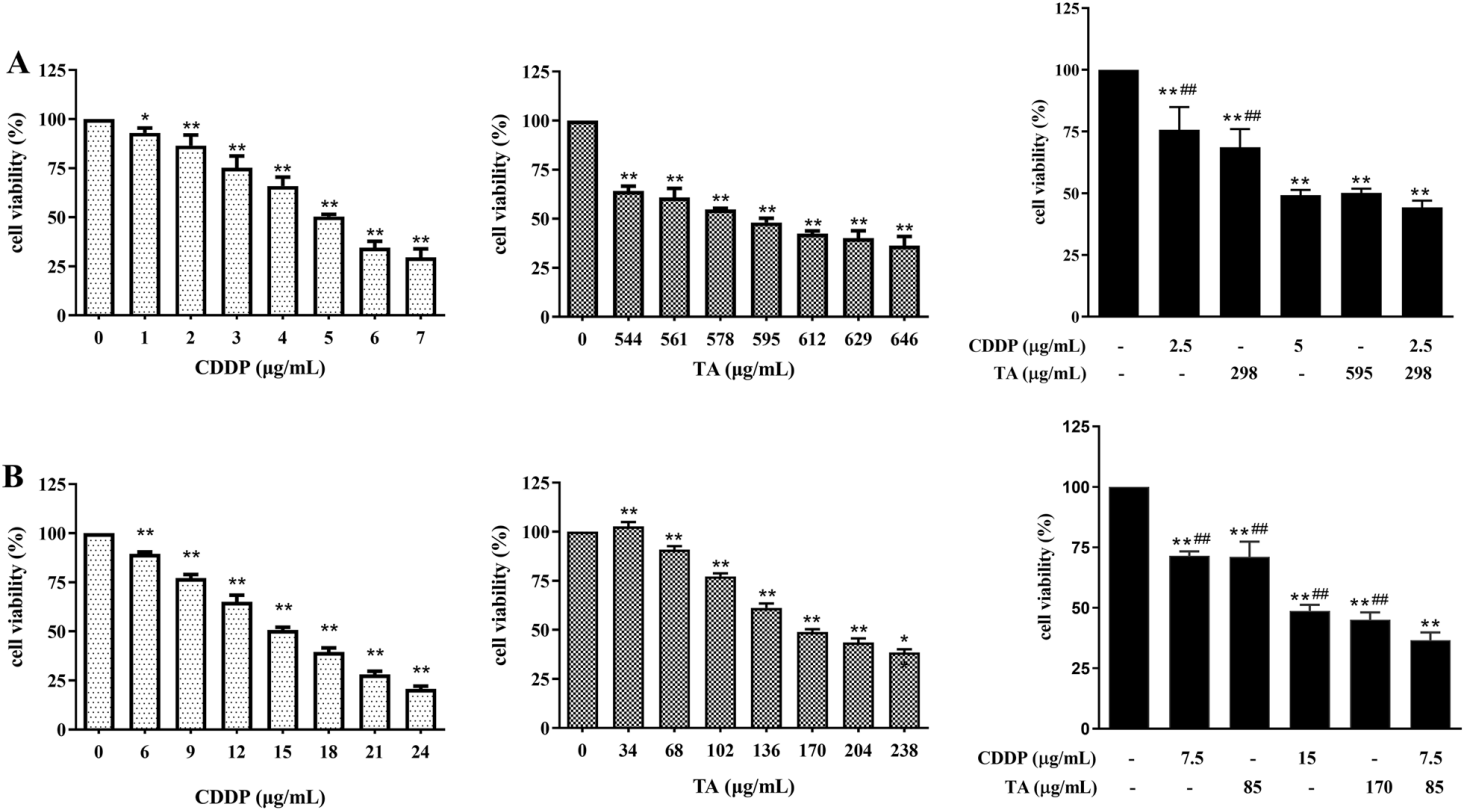
CDDP and TA inhibited GLC-82 and H1299 cell growth in vitro. GLC-82 (**A**) and H1299 cells (**B**) were treated with CDDP, TA, CDDP + TA for 24 h. MTT analysis indicated that CDDP and TA inhibited Lung cancer cell growth in a dose-dependent manner after 24 h, respectively. A combination of CDDP and TA synergistically inhibited cell growth. Experiments were performed in triplicate. *P < 0.05 and **P < 0.01, untreated control group vs. the TA- or CDDP-treated group, respectively; ^##^P < 0.01, the TA- or CDDP-treated group vs. the TA + CDDP group

The effect of the combination of CDDP and TA was assessed using MTT assays. The results revealed that the combination significantly inhibited the viability of lung cancer cells in a dose-dependent manner, with IC50 values of 5 μg/mL at 595 μg/mL for GLC-82 cells (Fig. 1A) and 15 μg/mL at 170 μg/mL for H1299 cells (Fig. 1B). CI and DRI are commonly used to observe the synergistic effect of two drugs; therefore, the IC50 of the two drugs were selected in this study to investigate their synergistic effect in lung cancer cells. The results showed that the inhibitory rates of CDDP, TA, and CDDP + TA on GLC-82 and H1299 cells were 15.8 ± 0.19%, 23.5 ± 0.45%, and 55.7 ± 2.7%; and 28.5 ± 1.9%, 29.0 ± 6.3%, and 63.4 ± 3.2%, respectively. The inhibitory effect of CDDP + TA on these cells was significantly greater than CDDP and TA individually (*P* < 0.01). At an inhibition rate of 20–56% and 13–70% in GLC-82 and H1299 cells, respectively, the CI was < 1 and the DRI was > 1 (Tables 2 and 3). These results indicated that the combination of CDDP and TA synergistically inhibited the growth of the two lung cancer cell lines, as well as reduced the effective dosage of the two drugs.

**Table 2:** Combined and dose reduction indexes of GLC-82 cells treated with CDDP and TA for 24 h

Growth inhibition ratio (%)	CI	CDDP			TA		
		C (μg/mL)		DRI	C (μg/mL)		DRI
		Single	Combined		Single	Combined	
20	0.64	2.4	1	2.40	481	106	4.53
28	0.71	3	1.25	2.40	513	148	3.45
50	0.76	5	2	2.50	595	212	2.80
56	0.94	5.5	2.5	2.20	612	298	2.06
70	1.15	7.7	4	1.93	673	425	1.58
77	1.38	9	5	1.80	717	595	1.21

CDDP: cisplatin; TA: tannin; CI: combined index; DRI: dose reduction index

**Table 3:** Combined and dose reduction indexes of H1299 cells treated with CDDP and TA for 24 h

Growth inhibition ratio (%)	CI	CDDP			TA		
		C (μg/mL)		DRI	C (μg/mL)		DRI
		Single	Combined		Single	Combined	
13	0.83	7	2.5	2.80	63	30	2.11
29	0.77	10	3.75	2.67	109	43	2.56
44	0.78	13	5	2.60	150	60	2.51
63	0.82	18	7.5	2.40	213	85	2.50
70	0.96	21	10	2.10	245	119	2.06
78	1.17	23	15	1.67	298	170	1.75

CDDP: cisplatin; TA: tannin; CI: combined index; DRI: dose reduction index

### TA-CDDP combination synergistically increases apoptosis in H1299 and GLC82 cells

**Fig. 2:**
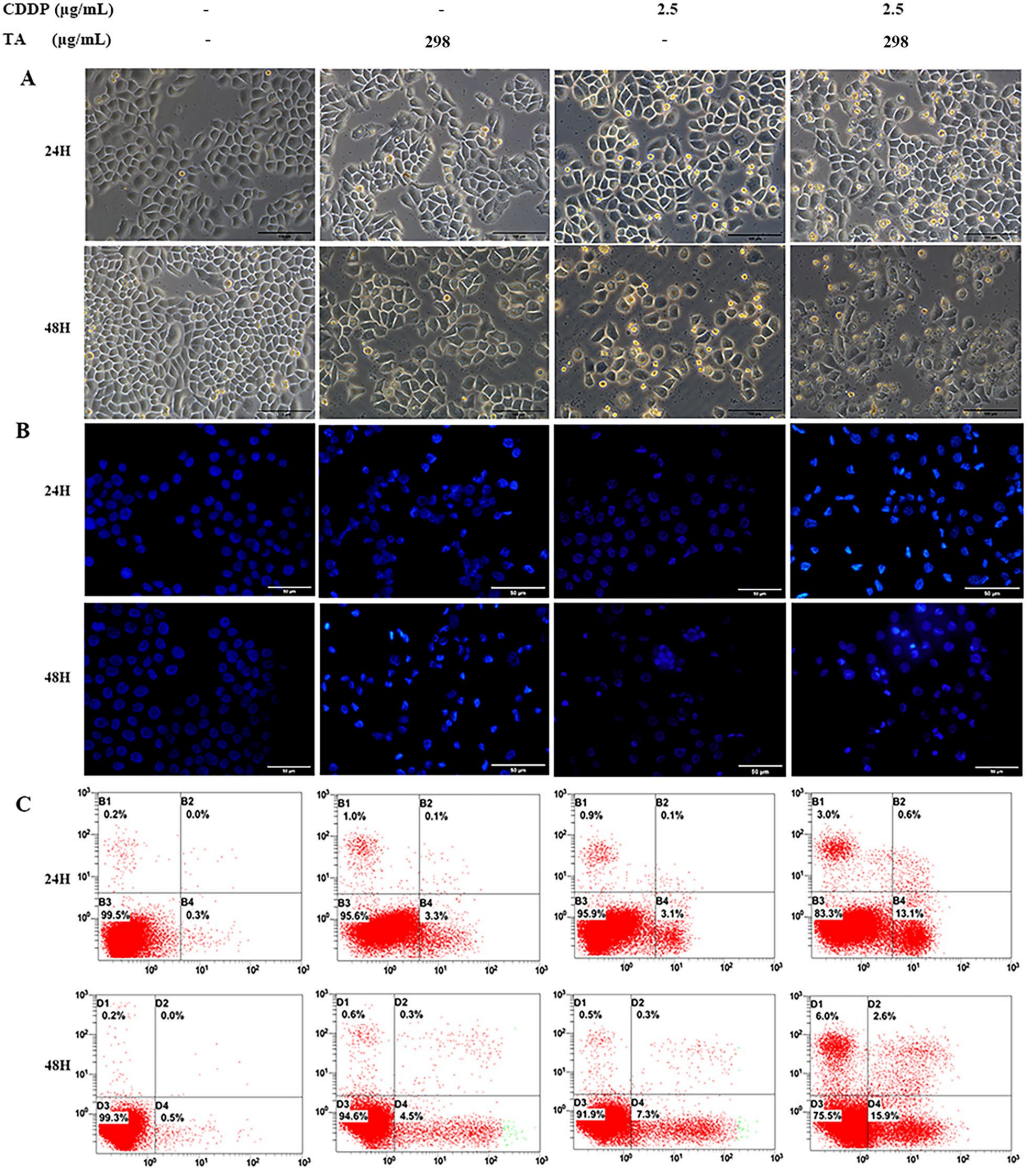
CDDP and TA synergistically increased the apoptosis of GLC82 cells. **A** The morphology of cells treated with CDDP or/and TA for 24 and 48 h was observed under a light microscope. The scale bar is 100 μm. **B** The nuclei of cells treated with CDDP and/or TA for 24 and 48 h were observed under a fluorescence microscope, and apoptotic bodies or nuclear debris were observed. The scale bar is 50 μm. **C** Flow cytometry analysis showed the percentage of necrotic cells, late apoptotic cells, viable cells, and early apoptotic cells (Denoted by quadrants D1, D2, D3, and D4)

**Fig. 3:**
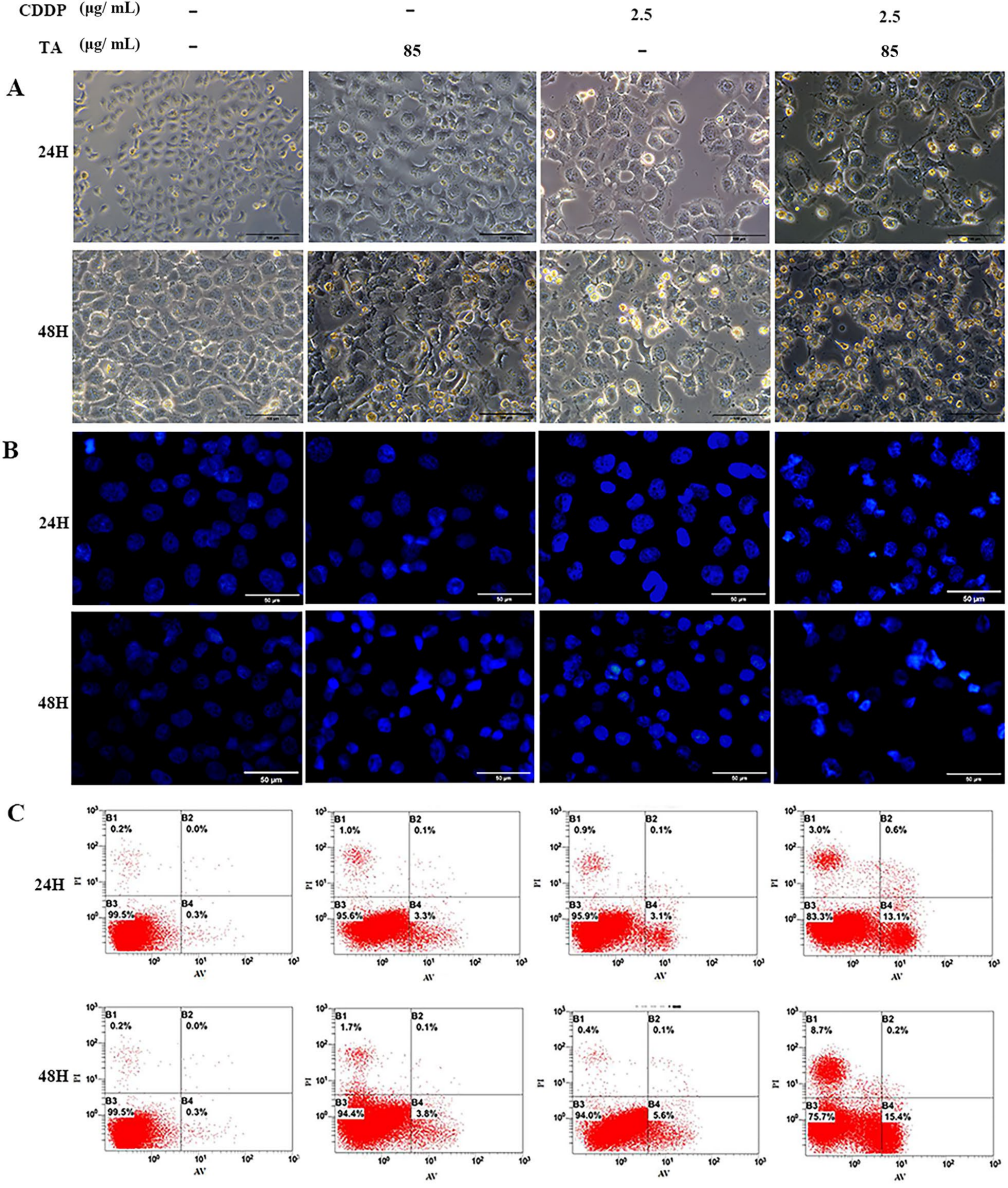
CDDP and TA synergistically increased the apoptosis of H1299 cells. **A** The morphology of cells treated with CDDP or/and TA for 24 and 48 h was observed under a light microscope. The scale bar is 100 μm. **B** The nuclei of cells treated with CDDP and/or TA for 24 and 48 h were observed under a fluorescence microscope, and apoptotic bodies or nuclear debris were observed. The scale bar is 50 μm. **C** Flow cytometry analysis showed the percentage of necrotic cells, late apoptotic cells, viable cells, and early apoptotic cells (Denoted by quadrants D1, D2, D3, and D4)

The effect of the drug combination on cell morphology was assessed under an inverted microscope. After 24 h of culture, GLC-82 cells in the control group were confluent and polygonal- or diamond-shaped with clear boundaries. In the monotherapy group, the cell volume decreased, cell spacing increased and the number of exfoliated cells and debris increased. This was more pronounced in the combined treatment group. After 48 h, the cell morphology of the control groups did not change significantly compared to that of the 24 h groups, while the cell volume in the CDDP + TA groups was significantly reduced, and the numbers of exfoliated cells and cell debris were significantly increased (Figs. 2A and 3A). These results indicate that the combination of CDDP and TA promoted apoptosis-like changes in the morphology of the two lung cancer cell lines.

DAPI staining is a commonly used method to observe nuclear morphological changes and cell apoptosis. After 24 h of culture, DAPI staining showed that the edges of the nuclei were smooth and complete, and the chromatin in the control group was uniform and light blue. However, after treatment with CDDP or TA, the nuclei was fragmented, and chromatin was concentrated; Nuclear pyknosis was considerably obvious, nuclear margin loss was severe, and more apoptotic bodies were observed in the CDDP and TA groups compared to the control. These effects were more pronounced in the CDDP + TA groups. After 48 h of culture, the nuclei of the control group did not change significantly compared to those at 24 h; however, nucleus fragmentation and aggregation were more prominent. After 48 h of treatment, the number of cells decreased significantly in the treated groups, and this effect was more pronounced in the combination group (Figs. 2B and 3B). These results indicate that both CDDP and TA can cause DNA damage; however, their combination synergistically enhanced DNA damage in lung cancer cells.

To further confirm that TA and CDDP synergistically increased the apoptosis of H1299 and GLC-82 cells, Annexin V-FITC/PI was used to detect early and late apoptotic cells, and the results showed that the apoptotic rate of GLC-82 cells treated with CDDP or TA for 24 h was 9.97 ± 0.75% and 5.5 ± 0.06%, respectively, whereas that of cells treated with CDDP + TA increased to 25.8 ± 1.5% (*P* < 0.01). After treatment with CDDP or TA for 48 h, the apoptotic rate of GLC-82 cells was 12.3 ± 0.44% and 10.5 ± 0.2%, respectively, and that of cells treated with CDDP + TA increased to 52.7 ± 1.4% (*P* < 0.01; Fig. 2C). For H1299 cells, the apoptotic rate at 24 h was 4.1 ± 0.1% and 4.1 ± 0.3% for the CDDP and TA groups, respectively; however, it significantly increased to 15.6 ± 1.6% in the CDDP + TA group (*P* < 0.01). After treatment with CDDP or TA for 48 h, the apoptotic rate of H1299 cells was 6.7 ± 1.0% and 5.8 ± 0.2%, respectively, and increased to 22.9 ± 2.1% after treatment with CDDP + TA (*P* < 0.01; Fig. 3C). The apoptotic rate in each treated group was increased at 48 h compared to that at 24 h. These results suggest that CDDP + TA had a greater proapoptotic effect on lung cancer cells than CDDP and TA separately, showing a synergistic enhancement effect.

### The combination of TA and CDDP activates the ER stress-mediated apoptotic pathway

**Fig. 4:**
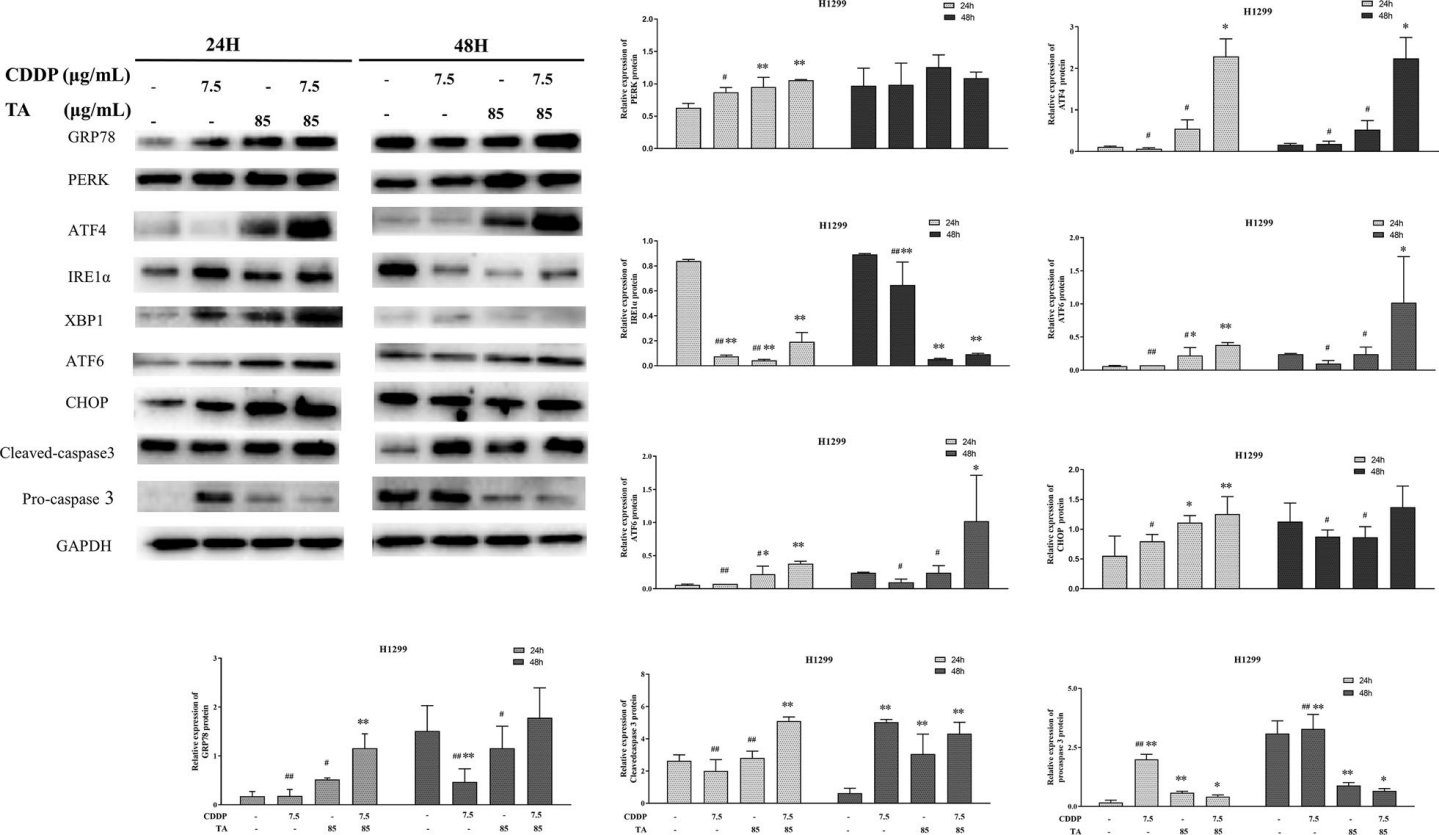
The expression of related factors by endoplasmic reticulum stress in CDDP and TA-treated H1299 cells. CDDP treatment combined with TA induced the expression of GRP78, CHOP, PERK, ATF4, IRE1α, XBP1, ATF6, CHOP, Cleaved-caspase3, Pro-caspase3 at the protein level in H1299 cells.

Preliminary experiments revealed that even if the dose was halved, the combination of CDDP and TA had a more noticeable effect on the apoptotic rate of lung cancer cells than single-drug treatments. CDDP can exert anticancer effects through various mechanisms, including DNA damage-induced apoptosis, mitochondrial apoptosis, and ER stress [23]. However, the mechanism of CDDP-induced ER stress remains unclear [8]. To determine whether the combination of CDDP and TA can cause ER stress-mediated apoptosis in lung cancer cells, we evaluated the expression levels of ER stress-related genes in our experimental groups using qRT-PCR and western blotting. GRP78 is a marker of ER stress [23] and plays an indispensable role in ER homeostasis. After 24 and 48 h of treatment in H1299 cells, the expression of GRP78 (Fig. 4A) in the CDDP + TA group was upregulated compared to that in other groups, indicating that the degree of ER stress caused by the drug combination was considerably higher than that caused by the drugs alone. However, if ER homeostasis is not restored, the activation of the UPR pathway may play an important role in ER stress-induced apoptosis by upregulating C/EBP homologous protein (CHOP) [24]. Therefore, we evaluated the expression of CHOP and found that it was upregulated in the combination drug group (Fig. 4B), and ER stress-induced apoptosis of lung cancer cells was increased.

**Fig. 5:**
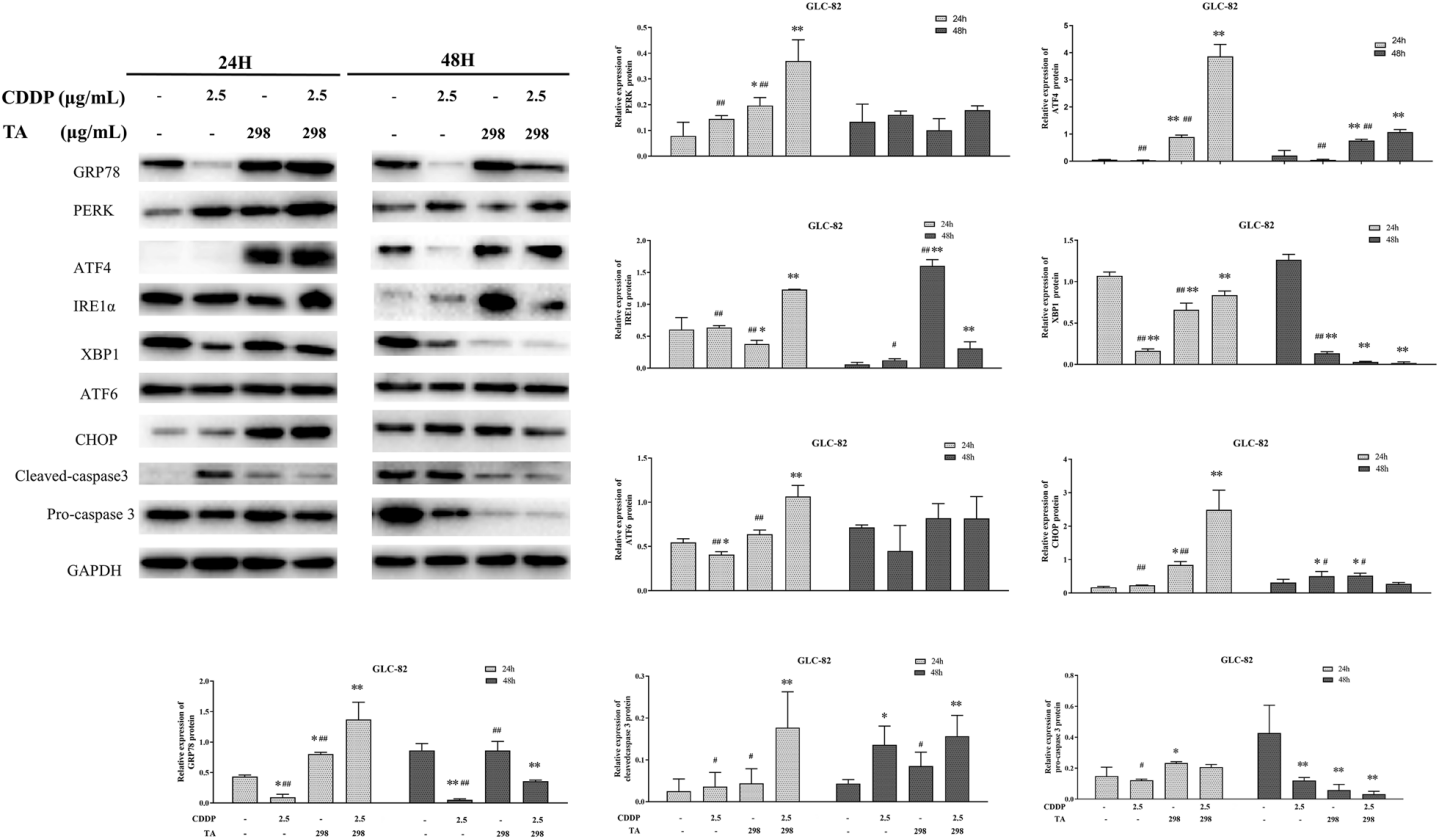
The expression of related factors by endoplasmic reticulum stress in CDDP and TA-treated GLC-82 cells. CDDP treatment combined with TA induced the expression of GRP78, CHOP, PERK, ATF4, IRE1α, XBP1, ATF6, CHOP, Cleaved-caspase3, Pro-caspase3 at the protein level in GLC-82 cells.

ER stress induces apoptosis in three classical pathways: IRE1α-XBP1 (inositol requiring enzyme l α-X box-binding protein 1), PERK-ATF4 (PKR-like ER kinase-activating transcription factor 4), and ATF6 (activating transcription factor 6). To investigate the mechanism by which the drug combination promotes ER stress-mediated apoptosis of lung cancer, we determined the gene and protein expression of three important factors in ER stress-induced apoptosis pathways after treatment. The protein levels of PERK, ATF4, and caspase-3 (Fig. 4) in H1299 cells were significantly upregulated in the CDDP + TA groups and were higher than those in the CDDP or TA groups. The qRT-PCR results were consistent with those of western blotting (data not shown). Surprisingly, the protein expression levels of PERK and caspase3 were extremely increased after 24 h of treatment than after 48 h of treatment. The gene and protein expression levels of IRE1α and XBP1 genes and proteins did not significantly change in the treatment groups compared to those in the control. Similar results were observed in GLC-82 cells, where the combination did not significantly affect the IRE1α-XBP1 pathway (Fig. 5). Moreover, the expression of ATF6 was increased after treatment with the drug combination. Therefore, the combined treatment of CDDP and TA in lung cancer cells may cause ER stress and induce apoptosis of tumor cells, mainly through the PERK-ATF4 and ATF6 pathways.

### Antitumor effects of TA in combination with CDDP in vivo

**Fig. 6:**
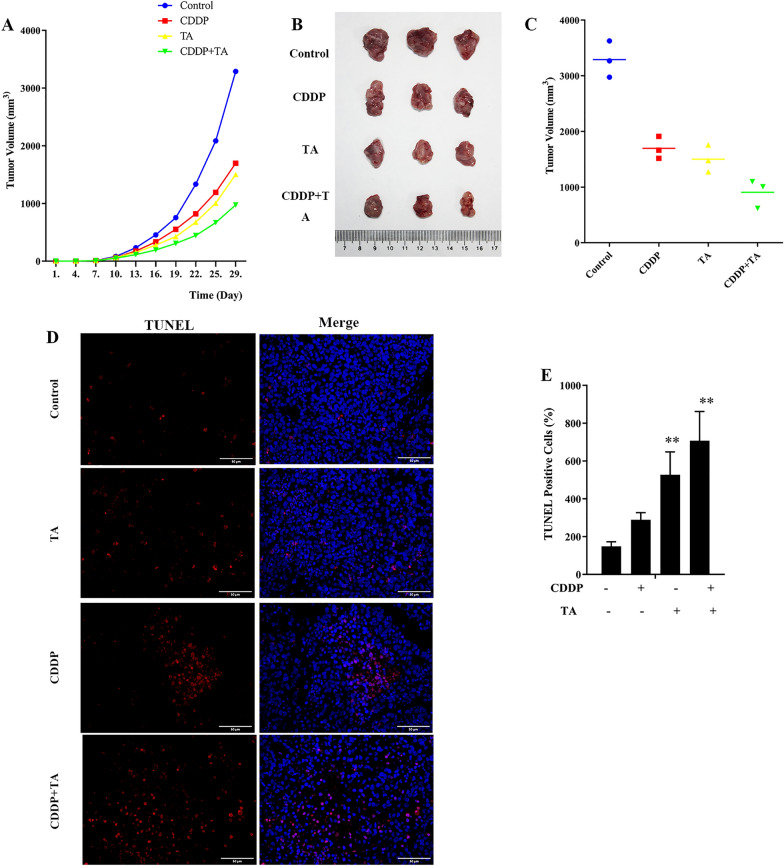
The effects of TA and CDDP on the growth of tumor xenografts. **A** Tumor growth curve treated with TA or CDDP. **B** as well as TA combined with CDDP was used to treat tumor volume in mice. **C** Quantitative analysis of tumor volume in mice treated with TA and CDDP. **D** TUNEL staining of tumor sections in mice treated with tannin and cisplatin (original magnification, *400). The red fluorescence represents the apoptotic cells, and the blue fluorescence represents the nucleus. **E** TUNEL staining was used to observe the apoptosis of tumor tissue sections

To verify the results obtained in vitro, H1299 cells were seeded subcutaneously into nude mice, followed by treatment with TA + CDDP. After intraperitoneal injections of the drugs, the body weight and tumor volume were measured every three days. On day 10, the tumor volume started to gradually increase (Fig. 6A), and on day 29, the mice were sacrificed, and tumor volumes were collected and measured. The tumor volumes in the control, CDDP, TA, and CDDP + TA groups were 3456.7 ± 179.5 mm^3^, 1657.3 ± 57.9 mm^3^, 1469.4 ± 91.1 mm^3^, and 921.1 ± 71.9 mm^3^, respectively (Fig. 6B, C). The tumor volume in the combined treatment group was significantly smaller than the other groups (*P* < 0.05). Therefore, although treatments with CDDP and TA individually inhibited xenograft growth, this effect was more pronounced with the combination of these drugs. Moreover, the TUNEL assay revealed that apoptotic cells were more abundant than normal living ones (Fig. 6D). Quantitative fluorescence analysis showed that the number of apoptotic cells was the highest in the combined-treatment group 708.00 ± 153.88, which was significantly higher than that in the control group 148.33 ± 24.19 (*P* < 0.05; Fig. 6E). These results suggest that CDDP combined with TA inhibit the growth of tumor cells and promote the apoptosis of tumor cells.

**Fig. 7:**
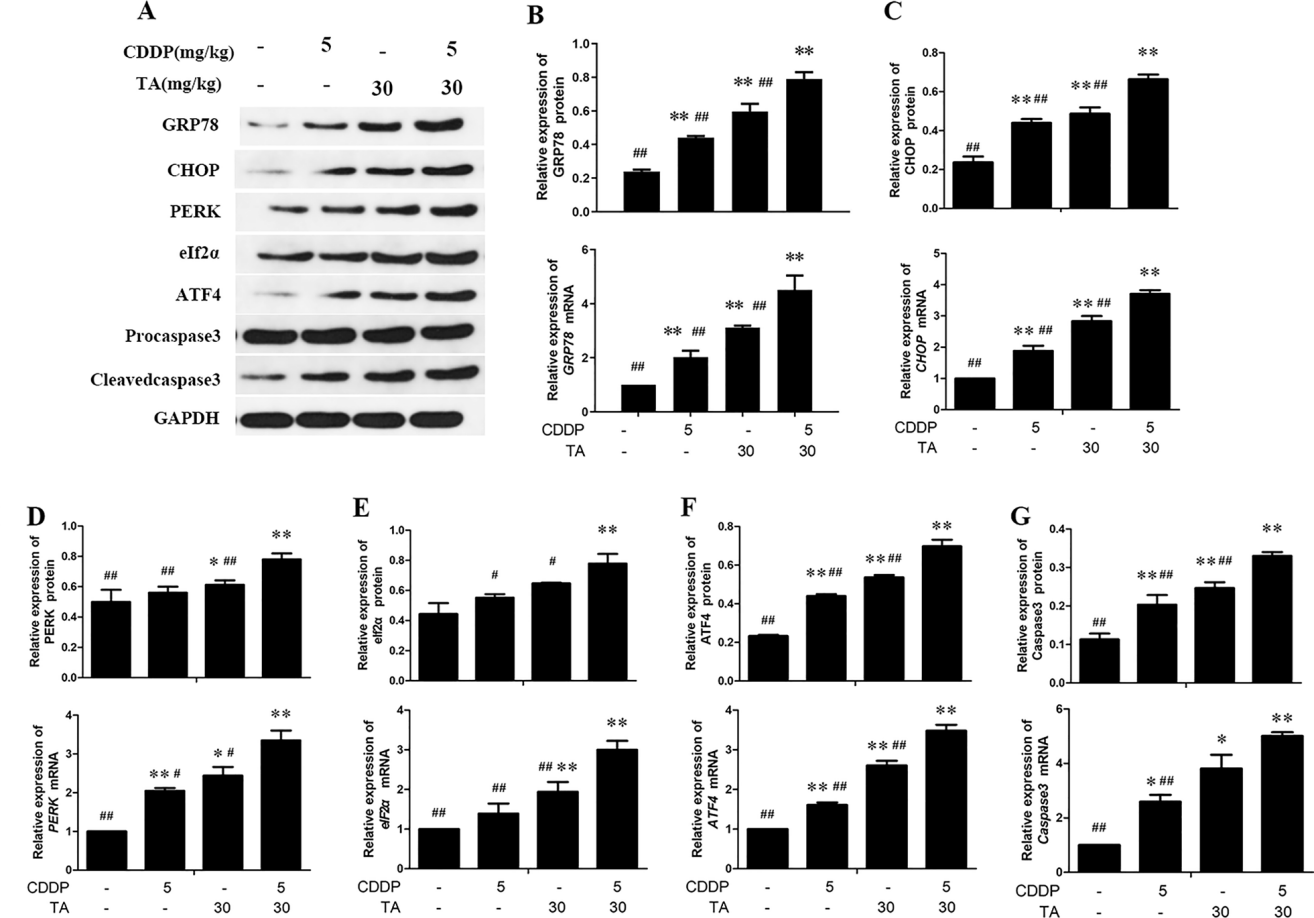
The expression of related factors by endoplasmic reticulum stress in tumor xenografts. Western blot analysed (**A**), that expression of HSP70 (**B**), CHOP (**C**), PERK (**D**) eIF2α (**E**) and Caspase3 (**G**) proteins and mRNA in tumor xenografts treated with CDDP and TA were assessed

To investigate whether CDDP + TA mediates apoptosis via the PERK-ATF4 pathway in vivo, the expression of key proteins and genes in the ER stress-mediated apoptotic pathway in the xenograft tumors was determined using PCR and western blotting. The results showed that in the CDDP + TA group, the mRNA and protein expression levels of GRP78, CHOP, caspase3, PERK, eIF2α, and ATF4 were 4.51 ± 0.54, 3.70 ± 0.12, 5.01 ± 0.14, 3.35 ± 0.26, 3.00 ± 0.22, and 3.48 ± 0.16; and 0.79 ± 0.06, 0.66 ± 0.04, 0.33 ± 0.01, 0.78 ± 0.05, 0.80 ± 0.09, 0.70 ± 0.05, respectively (*P* < 0.01; Fig. 7A–F). These protein expression levels were significantly increased in the CDDP + TA group compared to CDDP or TA groups (*P* < 0.05 and *P* < 0.01), and the apoptotic rate of transplanted tumor cells was significantly increased (*P* < 0.05 and *P* < 0.01). These results suggest that CDDP + TA inhibits the growth of xenograft mice via the ER stress pathway, PERK-ATF4, thereby promoting the survival of xenograft mice.

## Discussion

The clinical application of cisplatin, a widely used drug in the treatment of various tumors, is limited by its toxicity and the resistance of tumor cells to this drug. TA is a natural antioxidative drug that can scavenge free radicals and protect the body from oxidative cellular damage [16]. TA also inhibits the growth of various cancer cell types. In order to improve the clinical applicability of CDDP, it is often combined with other drugs to reduce its dosage and toxicity. Therefore, we investigated the effects of CDDP combined with TA. This study provides new and direct evidence that the combination of TA and CDDP synergistically inhibits lung cancer cell growth and promotes apoptosis. This combination reduced the required effective dose of CDDP in both GLC-82 and H1299 lung cancer cells, thereby reducing the toxicity and side effects of CDDP and enhancing its clinical practicability in treating lung cancer.

In this study, treatments with CDDP and TA alone inhibited the proliferation of human lung cancer cells (H1299 and GLC-82) in a dose-dependent manner. However, the combination of low doses of these two drugs induced a significantly higher rate of apoptosis than either of the drugs alone in H1299 and GLC-82 cells. This combination also effectively inhibited tumor growth in H1299 xenograft mice. Therefore, TA in combination with CDDP is a promising treatment for lung cancer.

ERS stress plays an important role in the maintenance of cellular homeostasis. This homeostatic state conforms to the "Yin-yang balance theory" of traditional Chinese medicine [25, 26]. ERS response keeps the functional balance of endoplasmic reticulum to maintain the normal function of cells and promote cell survival. If the stimulation is too long or too strong, the pathway of apoptosis is initiated, prompting cell death. Factors that promote cell survival are called "Yin", and factors that promote cell death are called "Yang". During the process of tumorigenesis, tumor cells are always in a hypoxic and hypotrophic environment due to excessive tumor growth and relatively backward angiogenesis. This environment triggers ERS and highly expresses GRP78 and GRP94 molecules, which is conducive to tumor survival [27]. As a result, tumor cells establish a new equilibrium and are always in a state of high levels of ER stress. If the level of ER stress is further increased by using an ER stress agonist, such as CDDP, TA, and other ERS agonist, High expression of the “Yang” factor of CHOP is upregulated highly molecule, accordingly, the cell balance will be broken and go to death [28].

ER stress plays an important role in cisplatin-induced apoptosis in lung cancer cells [29]. Several natural products exhibit antitumoral activity in lung cancer cells via ER stress-mediated pathways [30]. Omega-hydroxyyundec-9-enoic acid inhibits the viability of H1299 cells through ROS-dependent ER stress [31]. Natural terpenoid cantilever can cause mitochondrial dysfunction in NCI-H460 cells via ER stress and intracellular Ca^2+^ release [32]. Moreover, curcumin significantly increases the expression levels of CHOP and GRP78 in NCI-H460 through the release of intracellular Ca^2+^ [33]. We have previously shown that CDDP combined with TA enhanced the antitumoral ability of HepG2 cells against hepatocellular carcinoma [20]. This mechanism activates apoptosis by promoting the expression of PERK-ATF4 pathway-related molecules, which activates ER stress and subsequently induces apoptosis. Increased expression of GRP78, a marker of ER stress, enhances the sensitivity of tumor cells to anticancer drugs [34, 35]. Thus, GRP78 upregulation suggests the activation of the ER pathway. In this study, the results of in vitro and in vivo experiments showed that CDDP combined with TA increased the transcription and translation levels of GRP78, which were significantly higher than those in the groups receiving CDDP or TA alone. These results suggest that the combination of CDDP and TA promotes correct protein folding by increasing GRP78 expression, indicating that the ER stress pathway is activated. The PERK-ATF4 pathway is a classical ER stress pathway. Upon ER stress, PERK is self-expressed and dimerizes, and activated PERK phosphorylates the eukaryotic translation initiation factor 2α (EIF2α) subunit, which inhibits protein synthesis and increases the expression of downstream molecules ATF4 and CHOP [36, 37]. CHOP induces apoptosis by promoting the cleavage of caspase-3 [38], leading to PARP fragmentation, DNA damage, and nuclear fragmentation and concentration. Our experimental results showed that 24 h after treatment with the combination of CDDP and TA, the expression levels of key factor proteins and genes of the PERK-ATF4-CHOP axis and ATF6 in GLC-82 and H1299 cell lines were significantly upregulated compared to those in the control and single treatment groups. Moreover, another hub pathway, IRE1-XBP1 was not significantly changed. However, the degree of upregulation of some key factors was not significant after 48 h of drug combination treatment.

Cisplatin, a DNA chelator, induces apoptosis in cancer cells by binding to purine residues and causing DNA damage. Cisplatin can also induce apoptosis by inducing ER stress [29, 39]. The antioxidant TA scavenges excess mROS in cells and alters the expression of PERK, IRE1, and their regulatory proteins (ATF4, Bip, and PDI) to induce ER stress and apoptosis of cancer cells [40]. Therefore, the combination of CDDP and TA is most likely to act on the ER stress axis, PERK-ATF4-CHOP, leading to massive apoptosis in lung cancer cells.

Owing to its hydrophobic effect, TA has an ideal nanocarrier property because it can bind to drug molecules, forming a self-assembled cross-linked network [41]. Nanocarrier technology is often used to reduce drug doses and minimize the systemic side effects of chemotherapeutic drugs. In recent years, nanocarrier technology for delivering cisplatin has been found to enhance chemotherapy sensitivity and considerably reduce the side effects of cisplatin [7, 10, 41–43]. Therefore, future studies are required to investigate whether TA can be used as a nanocarrier for cisplatin to determine whether this delivery method can enhance the DNA destruction ability of cisplatin more than the combination of TA and cisplatin by standard intraperitoneal injections.

In conclusion, the combination of TA and cisplatin significantly promotes lung cancer cell apoptosis, and one of the synergistic antitumor mechanisms is mediated via the PERK-ATF4-CHOP pathway. These findings suggest a new adjuvant treatment for lung cancer.

## Data Availability

We declare that the materials described in the manuscript will be freely available to all scientists for non-commercial purposes.
